# Beyond communication: an update on transforming healthcare teams

**DOI:** 10.3389/fmed.2024.1282173

**Published:** 2024-02-21

**Authors:** Gabriela Fernández Castillo, Maha Khalid, Eduardo Salas

**Affiliations:** Making Effective Teams Laboratory, Department of Psychological Science, Rice University, Houston, TX, United States

**Keywords:** team science, team coaching, team-based curricula, simulation-based training, measurement

## 1 Introduction

In 2018, Salas et al. (1) offered 10 observations on the science of teams in healthcare. This perspective article offers a quick update, providing a new set of observations based on the latest findings. As a point-of-departure for these observations, we use one of medicine's most cited culprits of error: communication [see Table 1 for a complete list of articles discussing communication; see also Etherington et al. (2), Street et al. (3), Tiwary et al. (4)]—and our belief that while important, a sole focus on it fails to take a holistic approach.

**Table 1:** Articles included in the review of the literature.

**#**	**Authors**	**Title**	**Focus of research (*N* = 171)**	**Was communication discussed? (*n* = 153, ~89%)**	**Was communication noted as a source of medical error? (*n* = 41 ~28%)**
1	Cervantes-Sudio et al. (2021)	Are Filipino students ready to collaborate? Comparing the readiness of healthcare students for interprofessional education in the Philippines	Education on collaboration and teamwork (*n* = 41, ~24%)	Yes	Yes
2	Cerbin-Koczorowska et al. (2019)	As the twig is bent, so is the tree inclined: A survey of student attitudes toward interprofessional collaboration supported with the curricula analysis		Yes	No
3	Roberts et al. (2019)	Assessing students' and health professionals' competency learning from interprofessional education collaborative workshops		Yes	No
4	Oikawa and Donkers (2022)	Assessment of teamwork in interprofessional education		Yes	No
5	Kaifi et al. (2021)	Attitudes of nurses and physicians toward nurse–physician interprofessional collaboration in different hospitals of Islamabad–Rawalpindi region of Pakistan		Yes	No
6	Vincent-Onabajo et al. (2019)	Attitudes toward interprofessional practice among healthcare students in a Nigerian University		Yes	No
7	Flato et al. (2022)	Awareness of interprofessional learning as a tool to improve a Brazilian university curriculum		Yes	No
8	Watanabe et al. (2019)	Changes in attitudes of undergraduate students learning interprofessional education in the absence of patient safety modules: Evaluation with a modified T-TAQ instrument		Yes	Yes
9	Naumann et al. (2021)	Designing, implementing and sustaining IPE within an authentic clinical environment: The impact on student learning		Yes	No
10	Prill et al. (2022)	Determinants of interprofessional collaboration in complementary medicine to develop an educational module ‘complementary and integrative medicine' for undergraduate students: A mixed-methods study		Yes	No
11	Waltz (2020)	Determining the effectiveness of an interprofessional educational intervention for teamwork competencies among nursing, physical therapy, and pharmacy students		Yes	No
12	Naumann et al. (2021)	Designing, implementing and sustaining IPE within an authentic clinical environment: The impact on student learning		Yes	No
13	Caratelli et al. (2020)	Development and evaluation of an interprofessional seminar pilot course to enhance collaboration between health professions at a student-run clinic for underserved populations		Yes	No
14	Hammond and Morgan (2022)	Development of interprofessional healthcare teamwork skills: Mapping students' process of learning		Yes	No
15	Ganotice and Chan (2022)	Does collective efficacy drive readiness for interprofessional learning? Evidence from a large-scale interprofessional education program in Hong Kong		Yes	No
16	Clouder et al. (2022)	Education for integrated working: A qualitative research study exploring and contextualizing how practitioners learn in practice		Yes	No
17	Fenn et al. (2022)	Empathy, better patient care, and how interprofessional education can help		Yes	No
18	Huebner et al. (2021)	Establishing a baseline of interprofessional education perceptions in first year health science students		Yes	No
19	Gary et al. (2018)	Health science center faculty attitudes toward interprofessional education and teamwork		No	
20	Brewer and Flavell (2021)	High and low functioning team-based pre-licensure interprofessional learning: An observational evaluation		Yes	No
21	Raynault et al. (2021)	How interprofessional teams of students mobilized collaborative practice competencies and the patient partnership approach in a hybrid IPE course		Yes	Yes
22	Lairamore et al. (2018)	Impact of team composition on student perceptions of interprofessional teamwork: A 6-year cohort study		Yes	No
23	Chen et al. (2018)	Implementation, evaluation, and outcome of TeamSTEPPS in interprofessional education: A scoping review		Yes	No
24	Spaulding et al. (2021)	Interprofessional education and collaboration among healthcare students and professionals: A systematic review and call for action		Yes	No
25	Katoue et al. (2021)	Interprofessional education and collaborative practice in Kuwait: Attitudes and barriers from faculty		Yes	No
26	Machin et al. (2019)	Interprofessional education and practice guide: Designing ethics-orientated interprofessional education for health and social care students		Yes	Yes
27	Fenn et al. (2020)	Interprofessional education for complex neurological cases		Yes	No
28	Morrell et al. (2021)	Interprofessional Education Week: The impact of active and passive learning activities on students' perceptions of interprofessional education		No	
29	Winkler et al. (2021)	Interprofessional education workshop on aging: Student perceptions of interprofessional collaboration, aging, and cultural fluency		Yes	No
30	Seidlein et al. (2022)	Interprofessional health-care ethics education for medical and nursing students in Germany: An interprofessional education and practice guide		Yes	Yes
31	Browne et al. (2021)	Longitudinal outcomes of a brief interprofessional educational experience with or without an interprofessional education course		Yes	No
32	Roberts et al. (2018)	Perceived relevance mediates the relationship between professional identity and attitudes toward interprofessional education in first-year university students		Yes	No
33	Fox et al. (2018)	Teaching interprofessional teamwork skills to health professional students: A scoping review		No	
34	Brashers et al. (2020)	The ASPIRE model: Grounding the IPEC core competencies for interprofessional collaborative practice within a foundational framework		Yes	No
35	Keshmiri et al. (2021)	The effectiveness of interprofessional education on interprofessional collaborative practice and self-efficacy		Yes	No
36	Nyoni et al. (2021)	Toward continuing interprofessional education: Interaction patterns of health professionals in a resource-limited setting		Yes	Yes
37	House et al. (2018)	Medical student perceptions of an initial collaborative immersion experience		Yes	No
38	Botma and Labuschagne (2019)	Students' perceptions of interprofessional education and collaborative practice: Analysis of freehand drawings		Yes	No
39	Olander et al. (2018)	A multi-method evaluation of interprofessional education for healthcare professionals caring for women during and after pregnancy		No	
40	Kara et al. (2018)	An interprofessional patient assessment involving medical and nursing students: A qualitative study		No	
41	Harris et al. (2021)	An innovative interprofessional curricular model for diverse partners who team up to support behavior change in individuals with chronic disease		Yes	No
42	King and Shaw (2022)	“… breaks down silos”: Allied health clinicians' perceptions of informal interprofessional interactions in the healthcare workplace	General interprofessional care research (*n* = 82, ~48%)	Yes	No
43	Wei et al. (2020)	A culture of caring: The essence of healthcare interprofessional collaboration		Yes	No
44	Johnson and Mahan (2019)	A qualitative investigation into behavioral health providers attitudes toward interprofessional clinical collaboration		Yes	No
45	Wei et al. (2022)	A systematic meta-review of systematic reviews about interprofessional collaboration: Facilitators, barriers, and outcomes		Yes	No
46	Seaton et al. (2021)	Allied health professionals' perceptions of interprofessional collaboration in primary health care: An integrative review		Yes	No
47	Cutler et al. (2019)	Are interprofessional healthcare teams meeting patient expectations? An exploration of the perceptions of patients and informal caregivers		Yes	No
48	Ulrich et al. (2019)	Attitudes toward interprofessional collaboration in young healthcare professionals		Yes	Yes
49	Walton et al. (2020)	Clinicians' perceptions of rounding processes and effectiveness of clinical communication		Yes	No
50	Bjørkquist et al. (2019)	Collaborative challenges in the use of telecare		Yes	No
51	Fox et al. (2021)	Communication and interprofessional collaboration in primary care: From ideal to reality in practice		Yes	No
52	Garner et al. (2021)	Cross cultural team collaboration: Integrating cultural humility in mHealth development and research		Yes	No
53	Kannisto et al. (2021)	Daily functioning support—A qualitative exploration of rehabilitative approach in acute hospitalized care		No	
54	Haruta et al. (2018)	Development of an interprofessional competency framework for collaborative practice in Japan		Yes	Yes
55	Albarello et al. (2019)	Does Hub-and-Spoke organization of healthcare system promote workers' satisfaction?		Yes	No
56	Capari et al. (2018)	Dynamics of an orthopedic team: Insights to improve teamwork through a design thinking approach		Yes	Yes
57	Madsen et al. (2022)	Effectiveness of an interprofessional ambulatory care model on diabetes: Evaluating clinical markers in a low-income patient population		Yes	No
58	Neuhaus et al. (2022)	Emergence of power and complexity in obstetric teamwork		Yes	No
59	Hertel et al. (2019)	Engaging patients in primary care design: An evaluation of a novel approach to codesigning care		Yes	No
60	Pakkanen et al. (2022)	Ethical issues identified in nurses' interprofessional collaboration in clinical practice: A meta-synthesis		No	
61	Oblea et al. (2019)	Evaluation of clinical nurse transition program at US Army Hospitals		Yes	No
62	Kinnaer et al. (2022)	Evaluation of interprofessional care processes for patients treated with oral anticancer drugs		Yes	No
63	Heath et al. (2018)	Exchanging implements: The micro-materialities of multidisciplinary work in the operating theater		Yes	No
64	McNaughton et al. (2021)	Existing models of interprofessional collaborative practice in primary healthcare: A scoping review		Yes	No
65	Lam et al. (2018)	Exploring healthcare professionals' perceptions of the anesthesia assistant role and its impact on patients and interprofessional collaboration		Yes	Yes
66	Sukhera et al. (2022)	Exploring implicit influences on interprofessional collaboration: A scoping review		Yes	Yes
67	Waggie and Arends (2021)	Exploring interprofessional teamwork at a tertiary public hospital in South Africa		Yes	Yes
68	Papermaster and Champion (2021)	Exploring the use of curbside consultations for interprofessional collaboration and clinical decision-making		Yes	No
69	Bollen et al. (2019)	Factors influencing interprofessional collaboration between community pharmacists and general practitioners—a systematic review		Yes	Yes
70	Manspeaker et al. (2019)	Fostering interprofessional teamwork through an immersive study abroad experience		Yes	No
71	Sutherland et al. (2022)	Good working relationships: How healthcare system proximity influences trust between healthcare workers		Yes	No
72	Leonardsen et al. (2018)	Handovers in primary healthcare in Norway: A qualitative study of general practitioners' collaborative experiences		Yes	No
73	Bilodeau and Tremblay (2019)	How oncology teams can be patient-centered? Opportunities for theoretical improvement through an empirical examination		Yes	No
74	Thomas et al. (2019)	How pharmacy and medicine students experience the power differential between professions: ‘Even if the pharmacist knows better, the doctor's decision goes'		Yes	No
75	Sifaki-Pistolla et al. (2020)	How trust affects performance of interprofessional health-care teams		Yes	Yes
76	Walmsley et al. (2021)	Identifying practical approaches to the normalization of interprofessional collaboration in rural hospitals: A qualitative study among health professionals		Yes	No
77	McKay et al. (2021)	Impact of interprofessional embedding of physical therapy in a primary care training clinic		Yes	Yes
78	Farooqui et al. (2020)	Interpersonal communication, teamwork effectiveness, and organizational commitment in Pakistani nurses		Yes	Yes
79	Chew et al. (2019)	Interprofessional bedside rounds: Nurse-physician collaboration and perceived barriers in an Asian hospital		Yes	Yes
80	Ulrich and Breitbach (2022)	Interprofessional collaboration among sport science and sports medicine professionals: An international cross-sectional survey		Yes	Yes
81	Adamson et al. (2018)	Interprofessional empathy: A four-stage model for a new understanding of teamwork		Yes	No
82	Beaird et al. (2021)	Interprofessional rounding design features and associations with collaboration and team effectiveness		Yes	No
83	Bentley et al. (2018)	Interprofessional teamwork in comprehensive primary healthcare services: Findings from a mixed methods study		Yes	No
84	van Zijl et al. (2021)	Interprofessional teamwork in primary care: The effect of functional heterogeneity on performance and the role of leadership		Yes	No
85	Kvarnström et al. (2018)	Introducing the nurse practitioner into the surgical ward: An ethnographic study of interprofessional teamwork practice		Yes	No
86	Norful et al. (2022)	Mitigating primary care provider burnout with interdisciplinary dyads and shared care delivery		Yes	No
87	Hult et al. (2021)	Patient representatives: Crucial members of health-care working groups facing an uncertain role and conflicting expectations A qualitative study		Yes	No
88	Algahtani et al. (2021)	Perceptions and attitudes of different healthcare professionals and students toward interprofessional education in Saudi Arabia: A cross-sectional survey		No	
89	Rahman et al. (2019)	Perceptions of patient-centered care among providers and patients in the orthopedic department of a tertiary care hospital in Karachi, Pakistan		No	
90	Ylitörmänen et al. (2019)	Perceptions on nurse–nurse collaboration among registered nurses in Finland and Norway		Yes	Yes
91	Albassam et al. (2020)	Perspectives of primary care physicians and pharmacists on interprofessional collaboration in Kuwait: A quantitative study		Yes	Yes
92	Hickey et al. (2018)	Prospective health students' perceptions of the pharmacist role in the interprofessional team		No	
93	Schmutz et al. (2018)	Reflection in the heat of the moment: The role of in-action team reflexivity in health care emergency teams		Yes	No
94	Fernandez et al. (2020)	Revealing tacit knowledge used by experienced health professionals for interprofessional collaboration		Yes	No
95	Carroll et al. (2021)	Seeing what works: Identifying and enhancing successful interprofessional collaboration between pathology and surgery		Yes	Yes
96	Kämmer and Ewers (2022)	Stereotypes of experienced health professionals in an interprofessional context: Results from a cross-sectional survey in Germany		Yes	Yes
97	Chollette et al. (2022)	Teamwork competencies for interprofessional cancer care in multiteam systems: A narrative synthesis		Yes	No
98	Best et al. (2021)	Teamwork in clinical genomics: A dynamic sociotechnical healthcare setting		Yes	No
99	Brewer et al. (2020)	Teamwork, collaboration and networking: Self-reported behavioral change following pre-licensure interprofessional clinical learning		Yes	No
100	Rowan et al. (2022)	The impact of huddles on a multidisciplinary healthcare teams' work engagement, teamwork and job satisfaction: A systematic review		Yes	No
101	Katoue et al. (2021)	The perceptions of healthcare professionals about accreditation and its impact on quality of healthcare in Kuwait: A qualitative study		No	
102	Durand et al. (2022)	The role of gender, profession and informational role self-efficacy in physician–nurse knowledge sharing and decision-making		Yes	No
103	Sena and Liani (2020)	The role of relational routines in hindering transdisciplinary collaboration: The case of the setting up of a team in an Italian Breast Unit		Yes	No
104	Real et al. (2019)	The social logic of nursing communication and team processes in centralized and decentralized work spaces		Yes	Yes
105	Mitchell and Boyle (2021)	Too many cooks in the kitchen? The contingent curvilinear effect of shared leadership on multidisciplinary healthcare team innovation		Yes	No
106	Yamamoto et al. (2022)	Understanding interprofessional team delivery of patient-centered care: A qualitative secondary analysis		Yes	No
107	Schilling et al. (2022)	Understanding teamwork in rapidly deployed interprofessional teams in intensive and acute care: A systematic review of reviews		Yes	Yes
108	Rydenfält et al. (2019)	What do doctors mean when they talk about teamwork? Possible implications for interprofessional care		Yes	No
109	Hu et al. (2018)	Investigating student perceptions at an interprofessional student-run free clinic serving marginalized populations		Yes	No
110	Pinho et al. (2018)	Investigating the nature of interprofessional collaboration in primary care across the Western Health Region of Brasília, Brazil: A study protocol		No	
111	Assafi et al. (2022)	It's all about presence: Health professionals' experience of interprofessional collaboration when mobilizing patients with hip fractures		Yes	Yes
112	Karlsson et al. (2020)	Organizing for sustainable inter-organizational collaboration in health care processes		Yes	No
113	Wieser et al. (2019)	Perceptions of collaborative relationships between seven different healthcare professions in Northern Italy		Yes	No
114	Dahl and Crawford (2018)	Perceptions of experiences with interprofessional collaboration in public health nursing: A qualitative analysis		Yes	Yes
115	Hasan et al. (2018)	Physicians' perspectives of pharmacist-physician collaboration in the United Arab Emirates: Findings from an exploratory study		Yes	No
116	Jones et al. (2021)	Physiotherapy new graduate self-efficacy and readiness for interprofessional collaboration: A mixed methods study		Yes	No
117	Collins et al. (2021)	Self-efficacy and empathy development through interprofessional student hotspotting		No	
118	Forsagärde et al. (2021)	The dialogue as decision support; lived experiences of extended collaboration when an ambulance is called		No	
119	Burm et al. (2019)	Using a sociomaterial approach to generate new insights into the nature of interprofessional collaboration: Findings from an inpatient medicine teaching unit		Yes	Yes
120	Lee et al. (2021)	Understanding decision-making in interprofessional team meetings through interpretative repertoires and discursive devices		Yes	No
121	Karam et al. (2022)	Interprofessional collaboration between general practitioners and primary care nurses in Belgium: A participatory action research		Yes	No
122	Pomare et al. (2020)	Interprofessional collaboration in hospitals: A critical, broad-based review of the literature		No	
123	Schot et al. (2020)	Working on working together A systematic review on how healthcare professionals contribute to interprofessional collaboration		Yes	No
124	Bajwa et al. (2020)	Intra versus interprofessional conflicts: Implications for conflict management training		Yes	Yes
125	Keller et al. (2019)	Disruptive behavior' in the operating room: A prospective observational study of triggers and effects of tense communication episodes in surgical teams	Measurement (*n* = 13, ~ 8%)	Yes	Yes
126	Khoshab et al. (2019)	A survey on teamwork status in caring for patients with heart failure: A cross-sectional study		Yes	No
127	Bajwa et al. (2023)	Development and validity evidence for the intraprofessional conflict exercise: An assessment tool to support collaboration		Yes	Yes
128	Jaruseviciene et al. (2019)	Development of a scale for measuring collaboration between physicians and nurses in primary health-care teams		Yes	Yes
129	Peltonen et al. (2020)	Instruments measuring interprofessional collaboration in healthcare – a scoping review		Yes	No
130	O'Neill et al. (2018)	Team dynamics feedback for post-secondary student learning teams		Yes	No
131	O'Neil et al. (2020)	Team dynamics feedback for post-secondary student learning teams: Introducing the ‘Bare CARE' assessment and report		Yes	No
132	Ganotice et al. (2022)	To IPAS or not to IPAS? Examining the construct validity of the Interprofessional Attitudes Scale in Hong Kong		Yes	No
133	Etherington et al. (2021)	Measuring the teamwork performance of operating room teams: A systematic review of assessment tools and their measurement properties		Yes	Yes
134	Blumenthal et al. (2022)	Development of a questionnaire to assess student behavioral confidence to undertake interprofessional education activities		Yes	No
135	Sicks et al. (2022)	Measuring interprofessional education and collaborative practice competencies: A content validity study of the Jefferson Teamwork Observation Guide^®^		Yes	No
136	Wooding et al. (2020)	Evaluation of teamwork assessment tools for interprofessional simulation: A systematic literature review		Yes	No
137	O'Neill et al. (2018)	A taxonomy and rating system to measure situation awareness in resuscitation teams		Yes	No
138	Cunningham et al. (2018)	Interprofessional education and collaboration: A simulation-based learning experience focused on common and complementary skills in an acute care environment	Simulation-based training (SBT) (*n* = 14, ~8%)	Yes	No
139	Connolly et al. (2022)	A narrative synthesis of learners' experiences of barriers and facilitators related to effective interprofessional simulation		Yes	Yes
140	Stehlik et al. (2018)	Effect of hospital simulation tutorials on nursing and pharmacy student perception of interprofessional collaboration: Findings from a pilot study		Yes	No
141	Register et al. (2019)	Effect of interprofessional (IP) faculty development on perceptions of IP collaboration and on IP behaviors		Yes	No
142	Jakobsen et al. (2018)	Examining participant perceptions of an interprofessional simulation-based trauma team training for medical and nursing students		Yes	No
143	Wai et al. (2021)	Exploring the role of simulation to foster interprofessional teamwork among medical and nursing students: A mixed-method pilot investigation in Hong Kong		Yes	No
144	Costello et al. (2018)	Student experiences of interprofessional simulation: Findings from a qualitative study		Yes	Yes
145	Hughes et al. (2021)	Trauma, teams, and telemedicine: Evaluating telemedicine and teamwork in a mass casualty simulation		Yes	No
146	Leithead et al. (2019)	Examining interprofessional learning perceptions among students in a simulation-based operating room team training experience		Yes	No
147	Villemure et al. (2019)	Examining perceptions from *in situ* simulation-based training on interprofessional collaboration during crisis event management in post-anesthesia care		Yes	Yes
148	Astbury et al. (2021)	High-fidelity simulation-based education in pre-registration healthcare programmes: A systematic review of reviews to inform collaborative and interprofessional best practice		Yes	No
149	Jowsey et al. (2020)	Performativity, identity formation and professionalism: Ethnographic research to explore student experiences of clinical simulation training		Yes	No
150	Laco and Stuart (2022)	Simulation-based training program to improve cardiopulmonary resuscitation and teamwork skills for the urgent care clinic staff		Yes	Yes
151	Chamberland et al. (2018)	The critical nature of debriefing in high-fidelity simulation-based training for improving team communication in emergency resuscitation		Yes	Yes
152	Baik et al. (2018)	Examining interprofessional team interventions designed to improve nursing and team outcomes in practice: A descriptive and methodological review	Team development intervention (TDI) (*n* = 20, ~12%)	Yes	No
153	Lumenta et al. (2019)	Quality of teamwork in multidisciplinary cancer team meetings: A feasibility study		Yes	No
154	Clapper et al. (2019)	A TeamSTEPPS^®^ implementation plan for recently assigned interns and nurses		Yes	No
155	Hendricks et al. (2018)	Fostering interprofessional collaborative practice in acute care through an academic-practice partnership		Yes	No
156	Weinstein et al. (2018)	Integration of systematic clinical interprofessional training in a student-faculty collaborative primary care practice		Yes	No
157	Junge-Maugh et al. (2021)	Key strategies for improving transitions of care collaboration: Lessons from the ECHO-care transitions program		Yes	Yes
158	Blakeney et al. (2019)	Purposeful interprofessional team intervention improves relational coordination among advanced heart failure care teams		Yes	Yes
159	Grant et al. (2018)	We pledge to improve the health of our entire community': Improving health worker motivation and performance in Bihar, India through teamwork, recognition, and nonfinancial incentives		Yes	No
160	Fox and Brummans (2019)	Where's the plot? Interprofessional collaboration as joint emplotment in acute care		Yes	No
161	Block et al. (2021)	A novel longitudinal interprofessional ambulatory training practice: The improving patient access care and cost through training (IMPACcT) clinic		Yes	No
162	Kuner et al. (2022)	Clinical outcomes of patients treated on the Heidelberg interprofessional training ward vs Care on a conventional surgical ward: A retrospective cohort study		No	
163	Zhang et al. (2021)	Developing interprofessional collaboration between clinicians, interpreters, and translators in healthcare settings: Outcomes from face-to-face training		Yes	Yes
164	Gregory et al. (2020)	Examining changes in interprofessional attitudes associated with virtual interprofessional training		Yes	Yes
165	Mink et al. (2021)	Impact of an interprofessional training ward on interprofessional competencies—A quantitative longitudinal study		Yes	No
166	Luo et al. (2022)	Relationships between changing communication networks and changing perceptions of psychological safety in a team science setting: Analysis with actor-oriented social network models		Yes	No
167	Vatnøy et al. (2022)	Associations between nurse managers' leadership styles, team culture and competence planning in Norwegian municipal in-patient acute care services: A cross-sectional study		Yes	No
168	Iachini et al. (2019)	Examining collaborative leadership through interprofessional education: Findings from a mixed methods study		No	
169	Willgerodt et al. (2020)	Impact of leadership development workshops in facilitating team-based practice transformation		Yes	No
170	Wu et al. (2018)	Promoting leadership and teamwork development through Escape Rooms		Yes	No
171	Körner et al. (2018)	A patient-centered team-coaching concept for medical rehabilitation		No	

Full references available upon request.

Upon surveying recent literature (i.e., 2018–2023), we found that 89% of articles discuss communication in some way, and 28% mention communication as one of the leading causes of medical error (5–8). However, in the following piece, our stance is that despite communication having been repeatedly cited as “the” medical culprit, it may not be the source of all contention (9). More recent findings identify other challenges, such as accountability (10), conflict management (11, 12), decision-making (13), reflecting on progress, and coaching as the primary challenges healthcare teams face (14). Moreover, communication is a multi-faceted competency that also requires a holistic view.

In our review, it was clear that research on interprofessional collaboration was alive and well (around 48% of articles fit in this general category; see Table 1). However, more specific areas of research on interdisciplinary collaboration emerged, pointing to four primary areas of development: interprofessional education (24%), team development interventions [TDIs, see Lacarenza et al. (15); 20%], simulation-based training (SBT; 8%), and lastly, measurement (8%). Together, these areas point to a growing attention on the team as a whole—rather than on a single competency (i.e., communication). Guided by the findings from Table 1 and other extant developments, we provide an update on the observations made by Salas et al. (1). Doing so highlights what the last 5 years have taught us.

**Figure 1:**
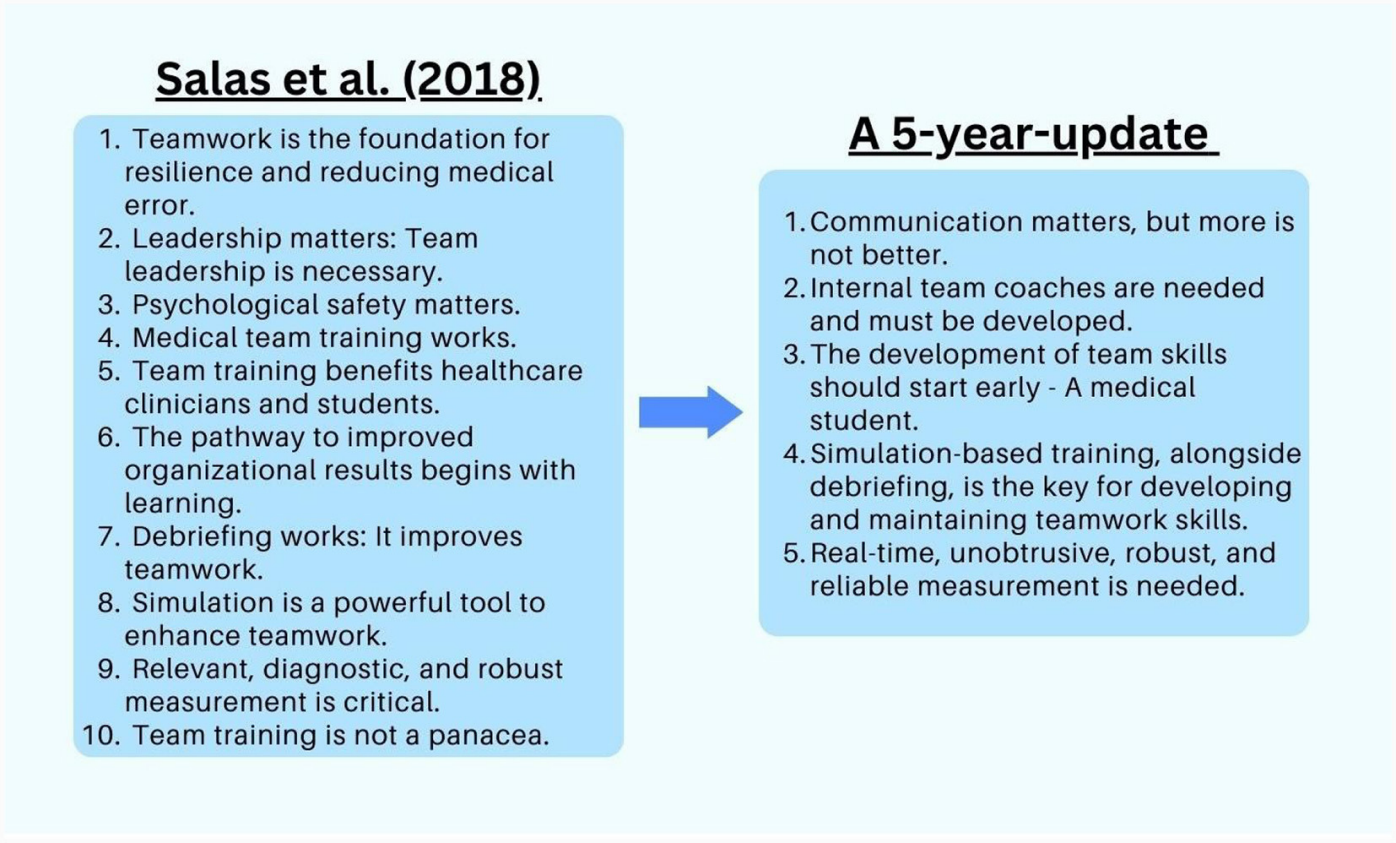
Holistic teamwork strategies.

In the following subsections, we discuss how these observations can continue to transform healthcare teams for the better and how they all work together to foster teamwork throughout healthcare practitioners' workplace lifespans. Figure 1 summarizes this update.

## 2 Observation 1: communication matters, but more is not always better

A plethora of teamwork competency frameworks exist. However, team scientists widely recognize that for teams to function effectively, they need to communicate [see Bollen et al. (16), who found communication is the most commonly reported influencing factor of collaboration]. Nevertheless, simply communicating is not enough, as meta-analytic evidence has shown that more is not better: better is better (17). In other words, the quantity of communication may not rectify teamwork issues. Quality is more indicative of better performance (17), and to have communication quality, teams need to ensure they have four things. Teams need to share unique information (such as a critical detail of a patient's history), have closed-loop communications (initiating, following up, and closing conversations), convey information when received (i.e., “I understand I need to be here in person for the meeting”), and make sure boundary spanners exist to communicate with individuals outside of the team (18). Understanding communication as simply sending information is an incomplete picture—and, as is now widely recognized, many environments are not conducive to it at first.

One of the most pivotal factors in ensuring communication *quality* is psychological safety—loosely defined as the ability to take intrapersonal risks [(19, 20); also see Keller at al. (12) and Luo et al. (21)]. In order to foster psychological safety and enable teams to speak up when necessary [a problem consistent in healthcare—where medical hierarchies persist, see Neuhaus et al. (22) and Seaton et al. (23)], teams need to engage in a variety of behaviors—involving specific implicit and explicit actions from clarifying expectations to promoting inclusivity [for a complete list of behaviors, see Kolbe et al. (24)]. Moreover, research has shown that healthcare teams must adapt, listen, and speak up properly and definitively amongst their colleagues and collaborators (25).

Therefore, not only is communication multi-faceted, but it is also one of multiple team competencies. For this reason, we argue that if healthcare professionals require all these skills, it is worth investing in strategies that target most teamwork competencies at once and let go of the idea that communication is a sole perpetrator of medical mistakes. This is not to say communication is unimportant, but simply that it is a piece of a larger puzzle. If an improvement in communication is not complemented by other teamwork competencies, teamwork as a whole is not likely to improve. In other words, good communication does not directly translate into good teamwork, and a more holistic approach is necessary.

## 3 Observation 2: internal team coaches are needed and must be developed

Team development interventions (TDIs) are designed with distinct purposes in mind. For example, there are training and process approaches that are necessary on different occasions (15). From our literature review, it became evident that there is a growing interest in testing and developing distinct types of TDIs in everything from leadership training (26) to process interventions (27). However, in the entirety of our review, only one article (27) touched upon what we thought to be a holistic TDI fitted to healthcare's immediate leadership needs: team coaching. Using extant supporting research, we provide rationale for its strength as an intervention and call attention to it as a great tool in nurturing teams in their lifespan.

Salas et al.'s (1) review [and more recently, Vatnøy et al. (28)] corroborated the importance of team leadership—which, as many have found (29), can help members coordinate their collective resources in accomplishing the team's work (27, 30). Furthermore, as the team coaching literature has evolved, it has been mostly conceptualized as a leadership strategy that both internal and external coaches can provide (31). Körner et al. (27) systematically developed a team coaching approach with the goal of leaving behind a team leader empowered enough to coach their teams. More recent advancements made by Maynard et al. (29) suggest that for healthcare, a profession with high power distance and a high degree of technical skills, internal coaches might be more successful in yielding improvements in team performance. This is because internal team coaches are experts in their field who understand immediate teamwork needs (31), allowing them to adapt with the team during times of need.

Körner et al.'s (27) team coaching approach and Maynard et al.'s (29) empirical study provided the primary advancements made in the last 5 years in team coaching in healthcare. Maynard and colleagues had a retired surgeon coach current surgeons, and these surgeons proceeded to utilize coaching skills on their surgical teams [Körner et al. (27) had a professional coach aid healthcare teams]. The results indicated that teams that underwent the intervention showed better surgical outcomes over those that did not (29). However, team coaching is supported by literature compromising the last 5 years and beyond, with fields like technology showing great potential in nurturing a team's overall health rather than a single aspect [e.g., Liu et al. (32); also see Fernández Castillo and Salas (33)]. Team coaching offers a powerful avenue to foster teamwork because it can tackle multiple teamwork competencies simultaneously (29, 33). We know that it can do three specific things (33): increase group effort (27, 32, 34), better interpersonal processes via improvements in psychological safety (19, 35), and lastly, increase team knowledge and learning (27, 36). By improving these things, general teamwork is improved. For example, if a team is failing because of a lack of information sharing (a facet of quality communication), improvements in group effort where people are encouraged to share can address this issue [see Körner et al. (27), who approach this issue via goal attainment]. Suppose a team is failing because people have information but do not feel safe speaking up. In that case, team coaching can alleviate this by creating a climate for safety where the internal leader establishes norms of respect regardless of medicinal hierarchies (33). We seek to highlight the fact that rather than focusing on communication or any other single teamwork competency, team coaching seeks to nurture team wellbeing as a whole and over time—leaving behind team leaders who can guide their teams without external intervention (27). Moreover, if implemented as more than a one-time intervention over the lifespan of healthcare practitioners' professional development, we could see other benefits, such as improving teamwork outcomes stemming from teaching leadership competencies in healthcare curricula (37). For this reason, we believe team coaching should be the avenue to fostering healthcare leaders, as by doing so, we simultaneously create a climate where teamwork is valued and fostered and where team members learn to communicate and *beyond*. We hope the next 5 years invest in team coaching as a TDI for leadership training, as gaps in the field (such as a lack of research with *ad-hoc* teams) are prominent and fruitful avenues of research.

## 4 Observation 3: the development of team skills should start early—A medical student

As observed in Figure 1, Salas et al. (1) offered the observations that team training works, benefits healthcare students and clinicians, and organizational results begin with learning. They noted that future research needed to focus on training sustainment and emerging teamwork modalities. This is more important than ever before, with some arguing that healthcare curricula as they stand today do not provide students with the competencies they need to be successful team players in the workplace (9, 14). Although training is an umbrella, we believe that team-based curricula can be a path to take, as they ensure that medical students have teamwork skills that are vastly important in healthcare (9).

During the last 5 years, the literature on team-based curricula and interdisciplinary education has flourished—representing a near quarter of identified literature (see Table 1). Most importantly, educators are continually encouraging the idea that healthcare education needs to be constructivist. Learners can take part in their own learning through methods such as SBT, which has shown to increase teamwork competencies (38, 39). Recent findings state that the most effective healthcare curricula are those that incorporate interprofessional simulation-based education [IPSE, Sezgin and Bektas (40)]. Alongside other types of interprofessional training (41), these methods provide students and clinicians the capability to learn to interact with healthcare professionals without compromising patient safety (42, 43). Moreover, the reason we believe team-based curricula is a holistic approach that allows students to grow in their teamwork abilities beyond and including communication—is that these methods provide students with social capital (44). As proposed by Burguess et al. (44), methods such as interprofessional team-based learning strategies allow students to build trust in their network, access and build resources such as knowledge and skills that each individual holds, and lastly, develop norms and rules for a team; which we believe can also aid in a team's coordination (18) and reflection capabilities (45). In other words, team-based curricula and interdisciplinary methods teach students to be well-rounded team members, not simply *communicators*. Though we do not believe team-based curricula is the end-all-be-all, a broad incorporation of team-based curricula can help healthcare practitioners develop teamwork competencies from the inception of their careers. This allows them to have built-in experience by the time they get to work on surgical teams, on research teams, and so on. Accompanied by other strategies, such as team coaching and continued SBT, it works to nurse teamwork competencies over time.

However, the literature has continued to emphasize that team-based curricula face the challenge that current healthcare structures do not support such interventions (46). While students like these approaches, some concerns are the lack of infrastructure for said interventions and the time required for implementing them (47). Notwithstanding, this should not dissuade hospitals, medical schools, and undergraduate institutions [see Kolbe et al. (48)] from aiming for an overhaul. While recent years have reiterated the challenge of incorporating these practices, the research continues to uncover that interprofessional methods yield significant results, such as improvements in shared decision-making and teamwork competencies (49) and improvements in clinical skills and a sense of belonging in the workplace (50). Curricula that take these reforming steps, such as incorporating TeamSTEPPS into healthcare students' education, have already shown promising results (51, 52). In addition to this, some medical schools are already incorporating these findings into their educational structures. One concrete example is the multimodal curriculum TeamFIRST, which aims to equip students with ten teamwork competencies necessary for team-based, interprofessional care.^1^ In this program, things like patient handovers are explicitly taught to students. TeamFIRST includes modules where students actively learn to communicate with their teams during handovers to improve patient safety. Students complete a simulated handover, practice sending and receiving information, and reflect on the experience to learn what can be improved.^1^ Such techniques have resulted in better handoffs in perioperative environments (46).

Overall, a multitude of research supports teamwork curricula's ability to show improvements, such as increasing student teamwork competencies (52). Therefore, the last 5 years have left us with the following takeaway: in a world that increasingly requires more interpersonal skills as technology fills in technical ones, systems and critical thinking are necessities that interdisciplinary team-based methods can provide (53). We believe that if we are to move forward with a focus on training sustainment as remarked by Salas et al. (1), we need strategies from beginning to end, and team-based education provides the first step in doing so.

## 5 Observation 4: simulation-based training, alongside debriefing, is the key for developing and maintaining teamwork skills

Salas et al. (1) stated that debriefing works, and simulation is a powerful tool to enhance teamwork. The last 5 years of research support these observations, with many studies remarking on how SBT should be incorporated alongside team-based curricula (40). SBT provides realistic clinical scenarios that closely mimic the challenges and complexities students encounter in their actual settings, enhancing the probability of transferring learned skills to real scenarios (54, 55). However, the core element of SBT lies in debriefings, which enable structured feedback and reflection, enhancing patient care by providing controlled, planned opportunities for facilitator training (56–59).

Recent developments show that SBT has successfully increased teamwork perception levels (60) and enhanced interprofessional collaboration in post-anesthesia care units (43). Moreover, simulation allows team members to undergo conflict in real-time, which could increase their conflict management skills (14). This training also allows teams to maintain teamwork skills over time (61) and improve attitudes toward teamwork (62). While we face the continuing challenge of refining methodological design (55), SBT (alongside debriefing) is a holistic approach that allows teams to face problems repeatedly and without risk. This targets more than one team competency, allowing members to develop trust with each other and allowing for more efficient team functioning.

In a field short on time, with team training and education often being set on the back burner, it is tempting to try and use one-time interventions. While these can yield some improvements (and are sometimes a necessity), if we are to tackle deep-rooted issues, we have to approach problems as what they are: a web instead of a needle in a haystack. Focusing on these evidence-based strategies allows healthcare practitioners to become more well-rounded team leaders and members. Team-based education supports teamwork competencies through a healthcare practitioner's workplace lifespan; SBT allows student and clinician teams to work and fail together without the fear of harming patients; debriefs allow them to discuss learnings; and internal team coaches foster teams in action, making for a system that supports teamwork every step of the way. However, in order to strengthen these strategies, the aid of real-time, unobtrusive, robust, and reliable measurement is needed.

## 6 Observation 5: real-time, unobtrusive, robust, and reliable measurement is needed

In relation to real-time, unobtrusive, robust, and reliable measurement in clinical practice, progress is being made. There are several methods that can be utilized that support ongoing assessment and feedback to improve patient care. Examples of effective methods include direct observations of clinical encounters (DOCEs), event-coding, entrustable professional activities (EPAs), and behavioral markers of specific observable behaviors or action that serve as indicators of proficiency in a particular skill or competency (63–66). However, as some note, assessment tools rely on the assumption that team measurement is equivalent to adding individual performance together (67). In order to continue advancing the science of teaming, we must move past this and look at team systems holistically. Recommendations include studying methods that examine the team system as a whole. One is the Team Emergency Assessment Measure (TEAM), an assessment that moves away from the summative assumption (67). Yet, we need more studies that study methods like TEAM in distinct clinical settings (as TEAM has only been examined in emergency settings) as a “one-size-fits-all” approach is not recommended.

Effective design of team-based strategies is closely tied to sound measurement practices like those mentioned above. Akin to blaming communication for medical error as a one-size-fits-all response, tailored measurement is frequently overlooked when designing team interventions. Though typical, this “one-size-fits-all” approach is misguided, as individuals operate in diverse contexts and take on tasks of varying complexities throughout their career trajectory. Measurement should be rooted in an evidence-based model that targets the specific context and clinical area being examined (68) while continuing to place the team where it belongs: an intricate and never-isolated system. The gap between research and practice is well-documented but remarked for a reason: teams exist in the wild and not in a laboratory setting.

Healthcare settings are highly controlled environments regarding personnel, procedures, and protocols. Learning and development can be enhanced in such complex settings when individuals are provided with real-time, unobtrusive, robust, and reliable feedback. While we recognize that this research is expensive and time-consuming, we must expand our understanding of measurement and be willing to take on the challenge that teams do not exist in isolation because measuring them as if they do provides limited opportunities for our science. The last 5 years have not provided a significant comprehensive strategy to address this problem—and it may be another five before there are any comprehensive strategies to discuss. However, by pivoting research to enhance our understanding of design measures related to team performance, we believe we can better diagnose a team's root issues instead of attributing errors to “communication gaps” in the field. For this reason, we recommend focusing on strategies that foster teams while continuing to develop measurement strategies that look at them in their real-time context. This could mean using strategies such as DOCEs and making sure they are accurately contextualized with clinical environments and team- and organizational-level factors.

## 7 The next 5 years

The last 5 years have highlighted the resiliency of the healthcare field over a pandemic, fluctuating demands, and mass technological change. Notwithstanding, such events have highlighted the need for new methods. With healthcare burnout at an all-time high (69, 70), as well as a lack of psychological safety in the field (71), we need methods that work together and nurse systems as a whole. It starts with teaching students to be team players, allowing them to practice, measuring teamwork robustly and reporting results accurately, and coaching teams throughout their life cycle. Effective teamwork in healthcare requires a holistic approach beyond a focus on communication. Moreover, we must understand that communication itself is multi-faceted, part of a system, and should be treated as such. To address these issues, we highlighted five observations that need further improvement but show extreme promise: higher *quality* communication, team coaching, team-based curricula, and SBT, and continued reliable measuring practices. By implementing these strategies and considering these observations, healthcare teams can work toward improving overall teamwork competencies and ultimately enhance patient care and outcomes.

## Author contributions

GF: Writing—original draft, Writing—review & editing. MK: Writing—original draft, Writing—review & editing. ES: Writing—original draft, Writing—review & editing.

## Footnotes

^1^Paquette S, Hernandez J, Preble R, Sadighi M, Kilcullen M, Hoffman O, et al. Team first: An innovative educational strategy for teaching teamwork competencies to health profession and medical students. (2023). Unpublished manuscript.
